# Morphological and functional changes in the rat retina associated with 2 months of intermittent moderate intraocular pressure elevation

**DOI:** 10.1038/s41598-018-25938-z

**Published:** 2018-05-16

**Authors:** Bingyao Tan, Akshay Gurdita, Vivian Choh, Karen M. Joos, Ratna Prasad, Kostadinka Bizheva

**Affiliations:** 1 Department of Physics and Astronomy, University of Waterloo, Waterloo, ON N2L3G1 Canada; 2 School of Optometry and Vision Science, University of Waterloo, Waterloo, ON N2L3G1 Canada; 3 Vanderbilt Eye Institute, Ophthalmology and Visual Sciences, Vanderbilt University Medical Center, Nashville, TN 37232 United States; 4 Department of Systems Design Engineering, University of Waterloo, Waterloo, ON N2L3G1 Canada

## Abstract

Morphological and functional changes in the rat retina and optic nerve head (ONH), associated with 8 weeks of intermittent moderately elevated intraocular pressure (IOP) were measured with a combined ultrahigh resolution optical coherence tomography (UHR-OCT) and electroretinography (ERG) system. The IOP of male Sprague-Dawley rats was raised in one eye to ~35 mmHg for 1 hour/day on 6 days each week using vascular loops. Single-flash ERG traces and volumetric UHR-OCT images of the ONH were acquired from both eyes before, during and after IOP elevations at weeks 1, 5 and 9 of the study. The UHR-OCT images showed depression of the posterior eye around the ONH during the IOP elevations, the magnitude of which increased significantly from week 1 to week 9 (p = 0.01). The ERG a-wave and b-wave amplitudes increased temporarily during IOP elevations and returned to normal ~30 minutes after loop removal. Recurrent intermittent IOP spikes caused > 30% decrease in the ERG a-wave and b-wave amplitudes measured during the IOP elevations over the course of 2 months. This study suggests that recurrent, relatively short-duration IOP spikes for extended period of time are associated with peri-ONH tissue hypercompliance and reduced retinal functional response to visual stimulation during acute IOP elevation.

## Introduction

Open angle glaucoma (OAG) is the second leading cause of blindness and in 2010 was estimated to have affected over 60.5 million people worldwide, with this number projected to reach over 100 million by 2040^1^. OAG is a chronic, multifactorial optic neuropathy, and in most cases is associated with elevated intraocular pressure (IOP), higher than 21 mmHg. So far, the exact mechanism of OAG pathology is still unknown, therefore, different experimental glaucoma models and methods have been developed to investigate the relationship between chronic high IOP exposure and OAG, including the DBA/2 J mouse^2^, laser photocoagulation of the trabecular meshwork^3^, anterior chamber cannulation^4^, injection of indocyanine green (ICG) dye into the anterior chamber combined with laser treatment^5^, injection of polystyrene microbeads to block the trabecular meshwork canals and to impede the aqueous humour flow in the anterior chamber^6,7^, episcleral vein injection of hypertonic saline^4,8^ and surgical circumlimbal suture^9–11^. All of these methods result in static IOP elevation, however, recent studies suggest that the IOP in human subjects changes dynamically over a 24 hour period, and severe IOP spikes and fluctuations are now considered important and independent factors of glaucoma progression^12,13^. Specifically, Zeimer *et al*.^13^ found that large IOP variation is associated with glaucoma risk and visual loss. A different study by Asrani *et al*. suggested that spiking IOP is a better diagnostic factor than mean IOP^14^; a headstand yoga posture (Sirsasana) was reported to elevate IOP immediately^15^, and routine practitioners could eventually develop glaucoma^16,17^. Moreover, recurrent uveitis and its treatment can cause intermittent IOP spiking that can lead to secondary glaucomatous damage^18^. As our knowledge of the relationship between dynamic IOP and OAG is still limited, a proper animal model mimicking diurnal IOP fluctuations or recurrent IOP spikes is needed. Joos *et al*.^19^ put an adjustable vascular loop anterior to the eyeball equator to elevate the IOP to 35 mmHg (1 h/day, 6 days/week over a period of 6 weeks), and reported thinning of the retinal nerve fiber layer (RNFL), retinal ganglion cell (RGC) somas loss, and axonal degeneration in the optic nerve in the treated eye. Gramlich *et al*.^20^ used corneal suction cup oculopression to raise the IOP to ~30–35 mmHg for one hour a day, and reported significant loss of RGC density in the treated eye after 30 repeated procedures over a period of 6 weeks. Both of these studies focused on quantifying morphological changes to the optic nerve head (ONH) caused by the chronically, intermittently elevated IOP by utilizing histopathology. However, the progressive alteration of the ONH morphology longitudinally over time is not readily accessible with single time-point histology, and whether there is any retinal dysfunction preceding the structural changes is also worth investigating.

Optical coherence tomography (OCT) is a non-invasive image modality that has been widely used for *in vivo* assessment of retinal and ONH structure in human subjects and animal models of retinal diseases^21^. It has sufficient axial and lateral resolution to resolve different retinal layers, and the scanning range is sufficient for capturing the volumetric structural changes of the ONH, superficial central retinal artery in rodents^22^ and deep lamina cribrosa in humans^23^ and monkeys^24^. Electroretinography (ERG) evaluates the retinal function by measuring changes in the retina’s electrical current evoked by visual stimulation. It is used in clinical and experimental studies to assess impaired retinal function, which can serve as a sensitive biomarker to IOP elevation-induced retinal damage. For example, acute IOP elevation studies^25–28^ confirmed that the retinal function recovers within several hours after normalization of the IOP, and the recovery rate is linearly related to acute IOP exposure^25^ and can be affected by age^27^. A combined OCT + ERG system provides the advantage of simultaneous measurement of the retinal morphology, blood flow and functional response to visual stimulation^29^, and as such can reduce variations in the OCT and ERG data caused by animal re-anesthesia and effect of cumulative IOP exposure rather than OCT and ERG measurements performed independently. In this study we utilized a combined OCT + ERG system, designed by our research group for measuring morphological and functional changes in the rat retina and ONH associated with intermittent, daily IOP elevations to a moderate level during a 2-month period.

## Methods

### Animals, anesthesia and IOP elevation

All experiments described here were approved by the University of Waterloo Animal Research Ethics Committee, were conducted in accordance with the Guidelines of the Canadian Council on Animal Care and conform to the ARVO Statement for the Use of Animals in Ophthalmic and Vision Research. Six 11-week-old male Sprague-Dawley rats (Harlan Laboratories Inc., Indianapolis, IN, USA), weighing ~350 g were used in our study. The animals were fed *ad libitum* and maintained in climate-controlled rooms with a 12-hour light/12-hour dark cycle. An adjustable vascular loop (Sentinal Loops; Sherwood-Davis and Geck, St. Louis, MO, USA), placed anterior to the equator of one eye, was used to elevate the IOP, following a procedure developed by Joos *et al*.^19^: IOP elevation targeted at 35 mmHg for one hour per day, 6 days per week over a period of 8 weeks. For the daily, short-term IOP elevation, the rats were placed in a custom, Broome-type rodent restraint. One drop of 0.5% proparacaine hydrochloride (Alcaine, Alcon, Mississauga, ON, Canada) was applied to the cornea prior to placement of the vascular loop and elevation of the IOP to 35 mmHg, and afterward every 10 min for the duration of the loop wear. The IOP was measured with a corneal rebound tonometer (Icare® Tonolab, Tuike, Finland) before placement of the vascular loop, immediately after loop placement, every 30 minutes during the loop wear, and at 1 min and 30 min after removal of the loop. Viscous lubricating eye drops (Liquigel, Allergan, Inc., Unionville, ON, Canada) were applied frequently to hydrate the cornea.

Morphological UHR-OCT images and single flash ERG were acquired on weeks 1, 5 and 9 of the study. On the experimental days, the rats were dark adapted for at least 12 hours before they were transferred in light impermeable cages to the research lab for the OCT imaging and ERG recordings. Only a dim, red headlight (631 nm, < 10.9 lux) was used by the researchers to manipulate the animals and the equipment during the experimental procedures and that light was switched off during the OCT and ERG data acquisition. The animals were placed on a heated custom animal holder with translational and rotational alignment capabilities to allow for alignment of the rat eye under the stationary OCT + ERG imaging probe. During the experimental procedures, the isoflurane level was kept at 2–2.5% and the animal vital signs, such as temperature, breathing rate, and heart rate were monitored every 10 minutes. Subcutaneous injections of 5 ml saline were administered immediately after anesthesia and every 1.5 hours throughout the experimental procedures to hydrate the animals. One drop 0.5% proparacaine hydrochloride (Alcaine, topical anesthetic, Alcon, Mississauga, ON, Canada) was applied to both eyes, followed by one drop of 0.5% tropicamide (Alcon, Mississauga, ON, Canada) for pupillary dilation. The IOP of the right eye was raised to 35 mmHg for 1 hour using the vascular loop and OCT and ERG recordings were acquired at 3 time points: immediately before the IOP elevation, after 40 minutes of loop wear and 30 minutes after loop removal. The UHR-OCT imaging and ERG recordings took ~20 minutes. The recording prior to IOP elevation in week 1 served as the baseline for this study, as the eyes were not exposed to any cumulative elevated IOP exposure at that time.

### Optical Coherence Tomography (OCT)

**Figure 1:**
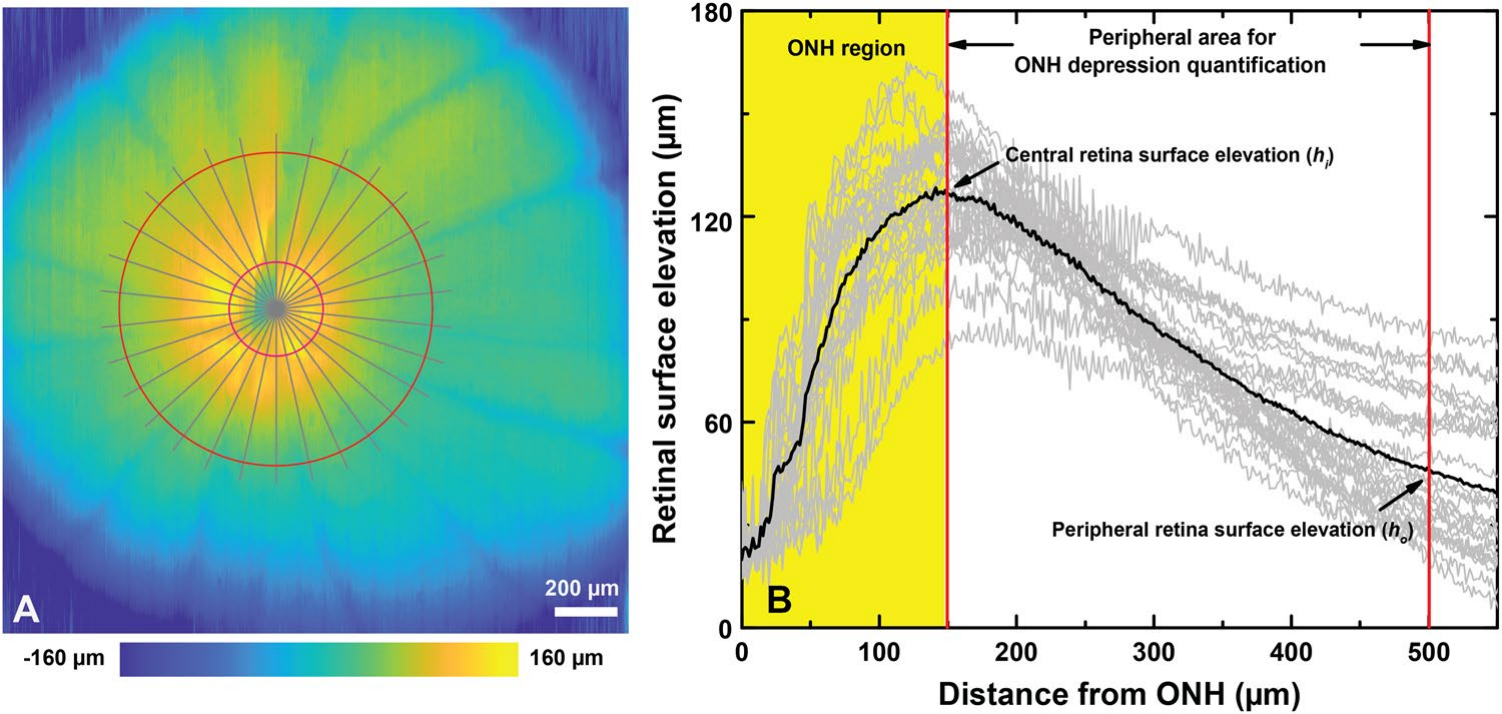
Quantification of the ONH depression. (**A**) A representative retinal surface map, where the red circles mark the central (h_i_) and peripheral (h_o_) boundaries, while the grey lines mark the radial cross-sections used for the ONH depression analysis. (**B**) Radial retinal surface elevations traces (grey lines) and an average retinal surface elevation trace (black line). The ONH depression was calculated as the average elevation difference between the central retinal surface elevation (h_i_) and the peripheral retinal surface elevation (h_o_).

Morphological images of the ONH were acquired *in vivo* with a research-grade, UHR-OCT system, that was developed by our research group for various animal retinal studies^29–31^. Briefly, the UHR-OCT system operates in the 1060 nm spectral region and provides ~3 µm axial and ~5 µm lateral optical resolution in the rat retina at an image acquisition rate of 92 kHz. The OCT imaging probe was designed to deliver a collimated imaging beam of 1.5 mm diameter and optical power of 1.7 mW to the rat cornea, resulting in ~5 µm lateral OCT resolution in the rat retina. The OCT imaging probe was integrated with a custom visual stimulator connected to the commercial ERG system (Diagnosys LLC, Lowell, MA)^29^. The visual stimulator was designed to focus the light from a white LED onto the pupil plane of the rat eye, thus generating a wide angle, almost uniform Maxwellian illumination of the rat retina. Detailed description of the optical design of the integrated OCT imaging probe and visual stimulator is included in a recent publication from our research group^29^. While under isoflurane anesthesia, a custom eyelid retractor was used to keep the imaged eye open. Artificial tears were administered every 5 minutes to keep the corneas hydrated. Volumetric OCT images (1000 A-scan x 1000 B-scan) of the ONH over an area of ~2 mm^2^ and circular OCT scans around the center of the ONH (Ø = 1 mm, 3000 A-scan) were acquired. A Labview based, *en face* preview display of the retinal surface was used for centering the ONH immediately prior to the acquisition of the volumetric and circular OCT scans. Cross-sectional and circular OCT images of the retina were generated from the one dimensional raw spectral data using a custom Matlab-based (Mathworks, Natlick, MA, USA) code. Volumetric UHR-OCT images of the rat retina were generated from the stacks of 1000 cross-sectional images by using commercially available software (Amira, FEI Visualization Sciences Group; and ImageJ software (http://imagej.nih.gov/ij/; provided in the public domain by the National Institutes of Health, Bethesda, MD, USA). A custom, Matlab-based sub-pixel registration algorithm was used to compensate misalignment between B-scans caused by eye motion. The retinal surface (inner limiting membrane) was segmented automatically by using a modified version of an algorithm proposed by Larocca *et al*.^32^, and a typical retinal surface elevation map is shown in Fig. 1A. The surface elevation profile was extracted radially from the center of the ONH (gray lines), which is manually selected as the converging point of the retinal major blood vessels. The ONH depression was calculated as the averaged elevation difference between the boundary of the ONH (at a distance of 150 µm from the center of the ONH) and a periphery reference (500 µm from the center of the ONH). The gray lines in Fig. 1B plot correspond to the radial retinal surface elevation for different radial cross-sections, while the average value for the retina is displayed as the black line. The difference between the central retina surface elevation (h_i_) and the peripheral retina surface elevation (h_o_) is defined here as the ONH depression. The nerve fiber layer + ganglion cell layer (NFL + GCL) and the ganglion cell complex (GCC) were segmented manually, and the regions containing large retinal blood vessels were excluded from NFL + GCL and GCC thickness calculation, in order to reduce the thickness variability due to the presence of blood vessels and the shadow artifacts associated with them.

The total retinal thickness was also evaluated by automatically segmenting the retinal OCT images from the inner limiting membrane (ILM) to the retinal pigmented epithelium (RPE). The thickness analysis was only carried out on the pre-loop measurements, in order to examine the effect of the chronic IOP elevation on the integrity of the inner and outer retina structure.

### Electroretinography (ERG)

ERG (Diagnosys LLC, Lowell, MA, USA) recordings were acquired sequentially from each eye. In addition to the positive loop electrode placed on the rat cornea, a negative electrode was placed in the skin behind the ear and a ground electrode was inserted into the scalp between the ears. The calibrated custom visual stimulator generated white light flashes of 7 ms duration and 1.14 log scotopic cd·s/m^2^ brightness^33^. Five ERG traces separated by 30 s intervals were acquired from each eye. The duration of each ERG recording was 1 s with a 500 ms pre-stimulus period. The ERG recordings were acquired following the order (pre-loop treated, pre-loop untreated, loop-on untreated, loop-on treated, post-loop untreated, post-loop treated). The amplitudes of the ERG a-wave and b-wave were measured as the maximum change in voltage from baseline to the first negative peak, and as the change in voltage from the a-wave peak to the peak positive change following the a-wave respectively. Implicit times for the ERG a- and b-waves, were measured from the onset of the light stimulus to the respective peak amplitude. Oscillatory potentials (OPs) were extracted from the ERG recordings using a Fourier bandpass filter (75–300 Hz) and the OP root mean square (RMS), a metric for the OP amplitude, was calculated for the time period between t = 20 ms and t = 70 ms post flash onset.

### Optic Nerve Histology

After the last IOP elevation, the rats were anaesthetized with isoflurane until they were unresponsive to toe pinches. Rats underwent cardiac perfusion with saline followed by 4% (w/v) paraformaldehyde in phosphate-buffered saline (PBS) and the optic nerves were harvested. Six-millimeter long segments of the myelinated optic nerve about 2 mm behind the globe were post-fixed in 1% glutaraldehyde and 4% paraformaldehyde in phosphate-buffered saline for 24 hours. Specimens were postfixed in 2% osmium tetroxide (Sigma-Aldrich, St. Louis, USA) for 1 hour and then embedded in Spurr’s low-viscosity embedding media (Electron Microscopy Sciences, Hatfield, PA). Semi-thin 700 nm sections were obtained and stained with 1% p-phenylenediamine (Sigma-Aldrich, St. Louis). A montage of the entire cross-sectional nerve was produced using a computer-driven motorized stage with a 100× oil-immersion objective (Provis AX70, Olympus, Melville, NY, USA). ImageJ software (http://imagej.nih.gov/ij/; provided in the public domain by the National Institutes of Health, Bethesda, MD, USA) was used to measure the nerve cross-sectional area. A fixed grid overlay was used to sample 20% of the total nerve cross-sectional area. All axons within the grid squares were manually counted by a masked person using ImageJ software to estimate axon density in the nerve (axons/mm^2^) and were categorized as “normal” or “degenerating” axons based upon their appearance. Normal axons were defined as possessing a defined myelin sheath surrounding clearly visible pale cytoplasm of the axon, and degenerating axons were defined as containing unraveling myelin sheaths or cytoplasm that were darkened or containing cellular debris. Total number of axons was estimated as the product of the mean axonal density and the nerve cross-sectional area following published protocols^34,35^.

### Axial eye length measurement

Because the loop wear causes deformation of the globe’s shape that can alter the amount of light from the visual stimulus that reaches the retina and therefore can affect the metrics of the ERG recordings, we conducted additional measurements to quantify this effect. A swept-source OCT system with a 7-mm long scanning range, originally developed by our research group for imaging the human anterior segment^36^, was used in this study to measure precisely the rat’s axial eye length at normal and elevated IOP. Since the optical path length in the rat eye is longer than 7 mm, the OCT images of the rat eye were wrapped around the OCT zero delay line and aligned in such a way that the corneal apex overlapped with the retinal pigmented epithelium. Therefore, the axial eye length was computed as 2× the distance from the top edge of the OCT B-scan to the location of the corneal apex.

### Statistical Analysis

*A priori* analysis on the pre-loop measurements of ONH depression and ERG metrics was carried out using analysis of variance (ANOVA) with Greenhouse-Geisser adjustment (epsilon values < 0.75). Repeated-measures ANOVA was used to detect significant differences as a function of weeks, eyes and loop procedures in all data collected from our study. Greenhouse-Geisser adjustment was applied for epsilon values < 0.75. Bonferroni-corrected multiple comparison post hoc tests were used for determining differences between measurement weeks and loop procedures. Differences were considered significant for p ≤ 0.05. All data are presented in the manuscript as mean ± standard deviation (SD) and in the figures as mean ± standard error (SE).

### Data Availability

The datasets generated during and analysed during the current study are available from the corresponding author on reasonable request.

## Results

### IOP elevations

**Figure 2:**
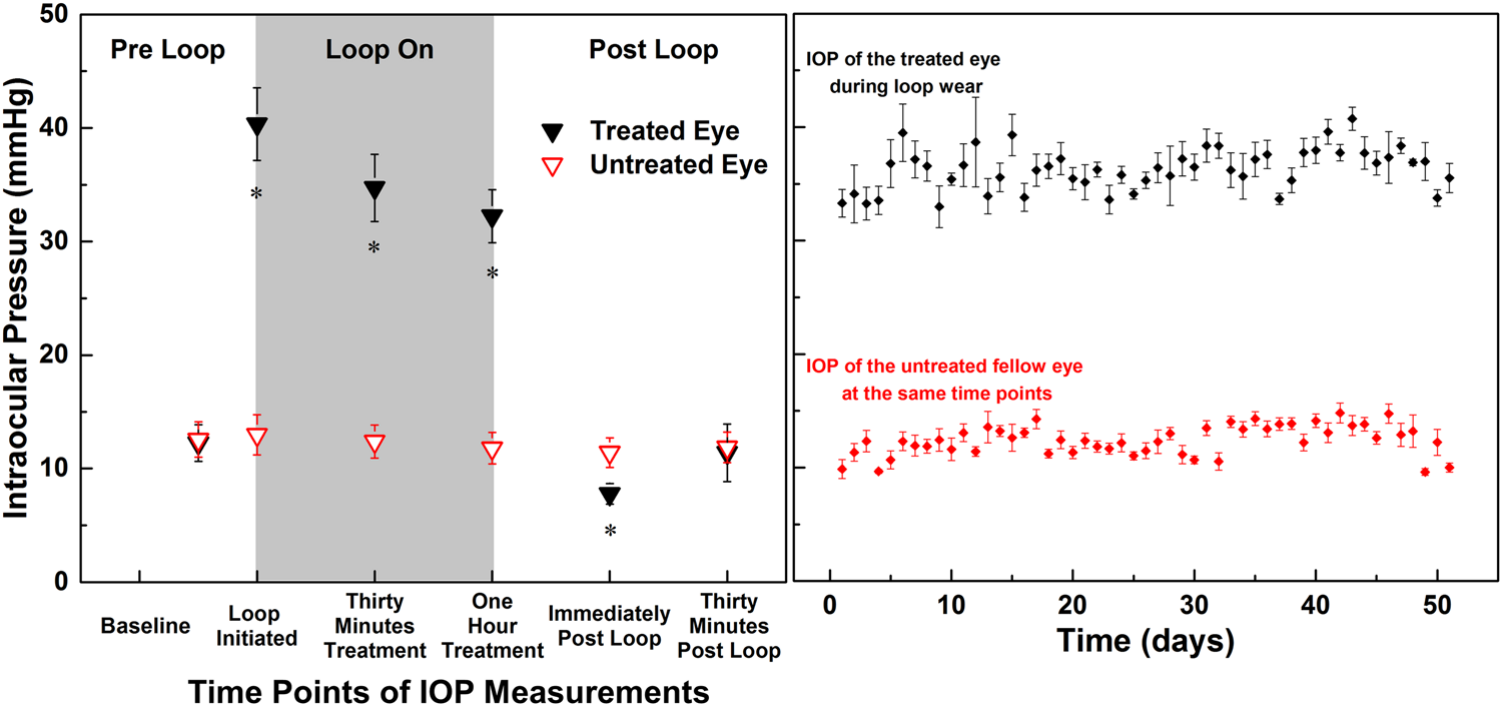
IOP data acquired during the ERG and UHR-OCT experimental procedures on weeks 1, 5 and 9 of the study are plotted for each measured time point (**A**), and mean + SE are plotted for the daily IOP elevation over the duration of the study. Each data point is an average of three measurements acquired from each rat. (**B**). *Significant differences between the treated and untreated eyes (P < 0.05).

The average pre-loop IOP was 12.5 ± 0.9 mmHg compared to 12.6 ± 1.0 mmHg of the untreated eye (p = 1.00). The IOP on the treated eye measured immediately after placement of the loop was 40.3 ± 1.1 mmHg and dropped to 34.7 ± 1.4 mmHg (p < 0.01), and 32.3 ± 0.8 mmHg (p < 0.01) after thirty minutes and one hour of loop wear respectively (Fig. 2A). The IOP of the untreated eye stayed fairly constant during loop wear (0 min: 13.0 ± 4.3 mmHg; 30 min: 12.4 ± 3.6 mmHg; 60 min: 11.8 ± 3.4 mmHg) (Fig. 2B). Immediately after loop removal, the IOP of the treated eye dropped to 7.8 ± 0.3 mmHg, a value significantly lower than the IOP of the untreated eye at the same time point (11.4 ± 0.5 mmHg, p < 0.01), and recovered to 11.2 ± 0.7 mmHg 30 minutes after loop removal. This was not significantly different from the IOP of the untreated eye (11.9 ± 0.6 mmHg, p = 1.00) (Fig. 2A).

### Retinal Thickness

**Figure 3:**
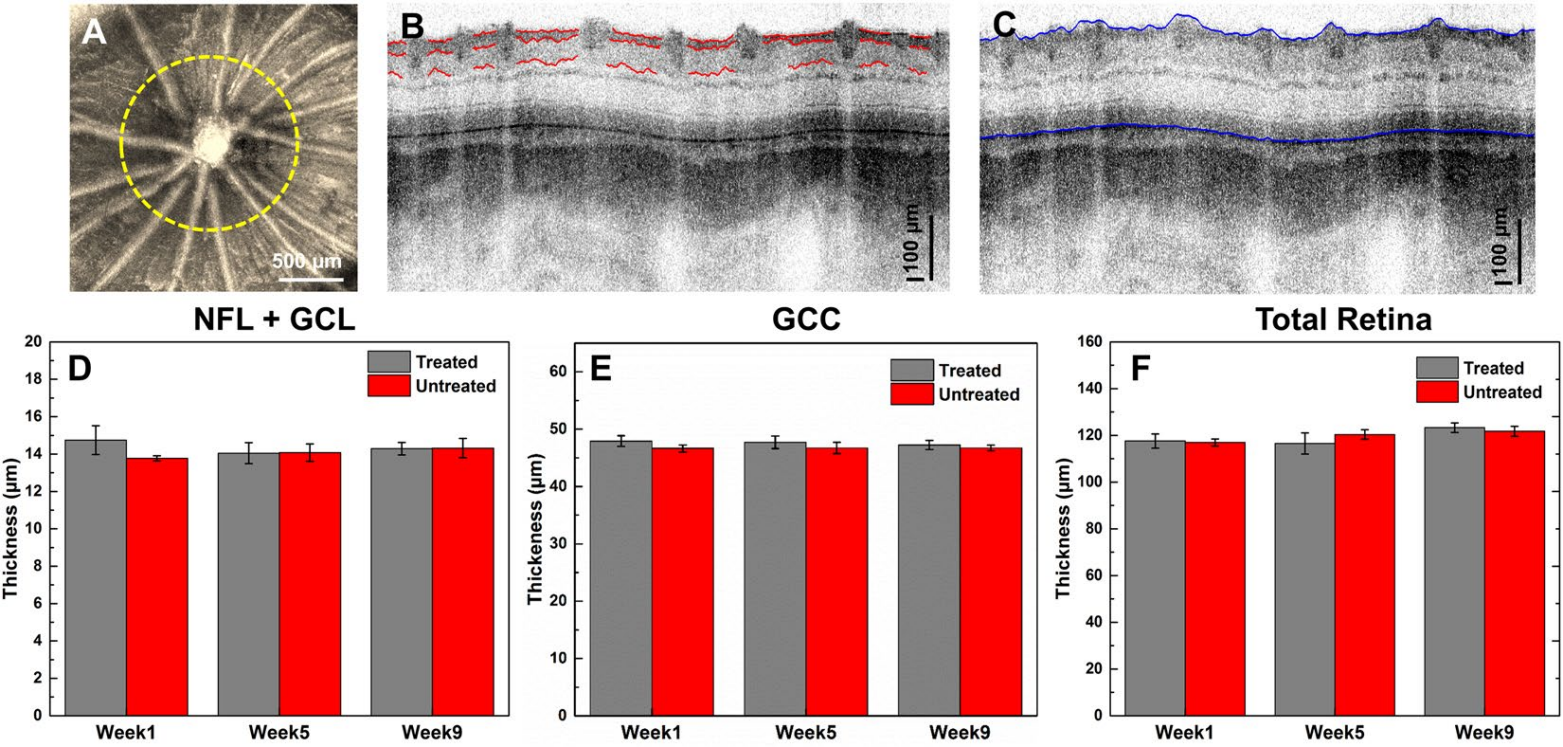
(**A**) Maximum projection enface OCT image of the rat retina, centered at the ONH. The yellow dashed line marks the OCT circular scanning pattern used to the retinal thickness evaluation. (**B**) Representative results from the manual segmentation of the NFL + GCL and (**C**) automatic segmentation of total retinal thickness. (**D**–**F**) Statistical results for the NFL + GCL, GCC and total retinal thickness.

Figure 3A shows a representative enface, maximum projection retinal image centered at the ONH, where the yellow dashed line marks the location of the circular OCT scan pattern. Figure 3B,C show the typical examples from the manual segmentation of the NFL + GCL and GCC layers, and the automatic segmentation of the total retinal thickness. Figure 3D,E show statistical data for the retinal thickness, measured at weeks 1, 5, and 9 for the duration of the study. There were no significant differences in all three thickness metrics (all p > 0.05), indicating that the chronic intermittent IOP elevation did not cause any significant changes in the retinal morphology that are measurable with UHR-OCT.

### UHR-OCT Morphology

**Figure 4:**
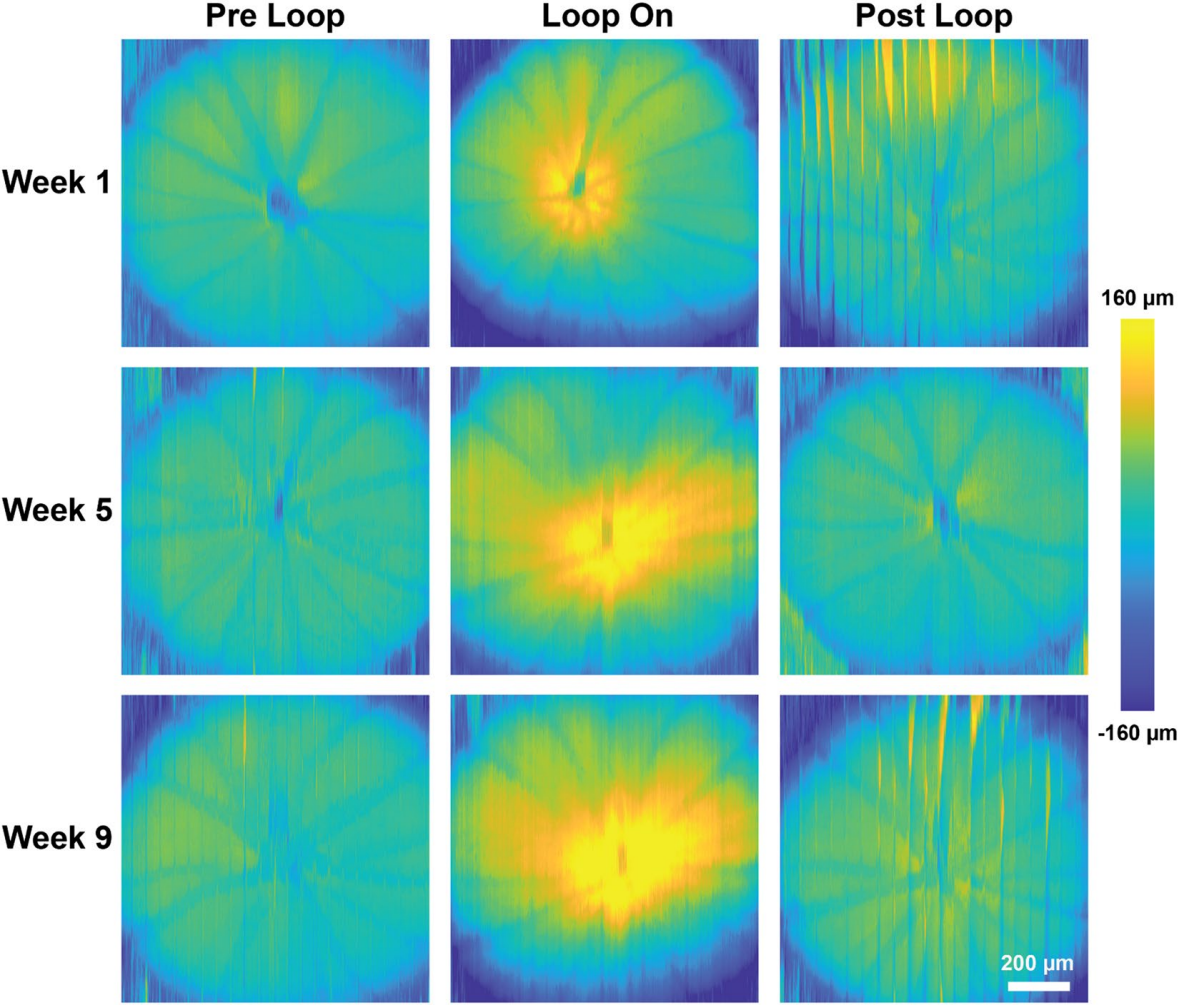
Representative retinal surface elevation maps acquired from the treated eye at baseline (week 1) and weeks 5 and 9 of the study. Vertical lines in the maps correspond to eye motion artifacts.

Figure 4 shows representative ONH elevation maps computed for the pre-, during- and post-loop measurements. The maps show that temporary IOP elevation is associated with backward bowing of the ONH and that within 30 min of loop removal, the shape of ONH recovers to a form similar to that of the pre-loop measurement. The vertical lines correspond to motion artifacts that could not be compensated well with image processing. Visually, there is a larger ONH depression associated with acute moderate IOP elevation in weeks 5 and 9 compared to that in week 1.

**Figure 5:**
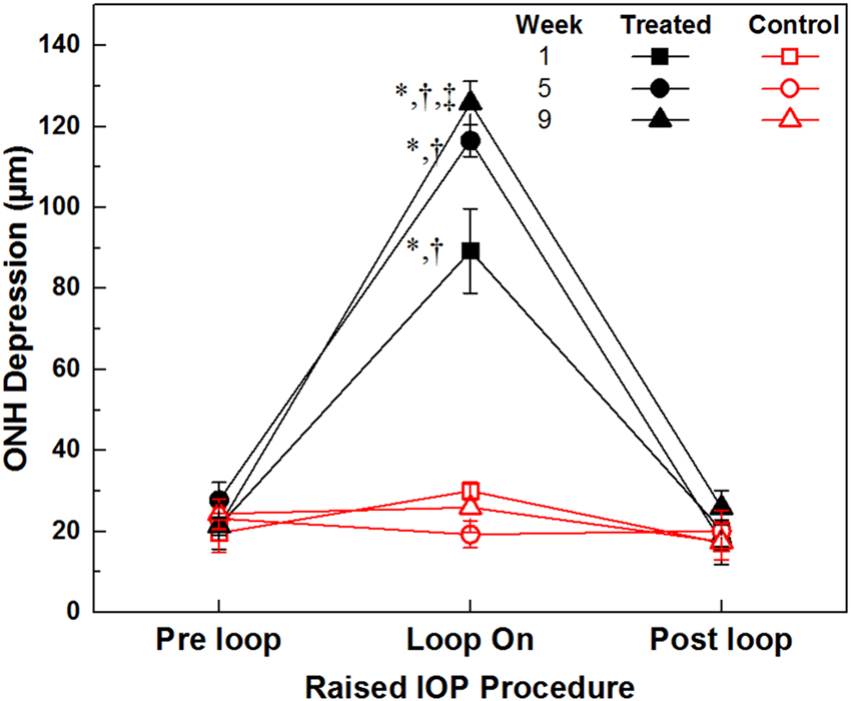
ONH depression as a function of IOP mean + SE. *Significant difference relative to respective pre-loop and week measurement (p < 0.05). ^†^Significant difference compared to the loop-wear measurements on the untreated eyes (p < 0.05). ^‡^Significant difference compared to the week 1 loop-wear measurements on the treated eyes (p < 0.05).

Figure 5 shows results from the quantitative analysis for the ONH depression in the treated and control eyes of 6 animals. *A* *priori* analysis of the pre-loop data showed that chronic, intermittent IOP elevation did not alter the pre-loop ONH depression significantly as a function of weeks and eyes (p = 0.42). However, during the loop-on measurements, the ONH depression increased progressively from week 1 to week 9 of the study during IOP elevation. At week 1, loop wear caused a relative ONH depression of 89.3 ± 25.6 µm compared to the pre-loop (21.4 ± 14.1 µm, p < 0.01) and post-loop values (20.3 ± 9.6 µm, p < 0.01), and no significant difference between pre-loop and post-loop conditions was detected (p = 1.00). Loop wear-associated, relative ONH depression measured at weeks 5 and 9 of the study increased by 131% and 140% respectively, compared to week 1 (week 1 vs week 5, p = 0.17; week 1 vs week 9, p = 0.01, week 5 vs week 9, p = 1.00). The ONH depression in the untreated fellow eye did not change significantly over time (all p = 1.00).

Analysis of the SS-OCT images showed 4.3 ± 0.9% increase in the axial length of the rat eye during IOP elevation to 35 mmHg, and returned to baseline 30 minutes after loop removal. No significant changes were observed in the anterior chamber depth or the iridocorneal angle.

### Electroretinograms

An *a priori* analysis was conducted on the pre-loop ERG measurements to examine the chronic intermittent IOP elevation effect on the basal retinal function. There is no interaction between eyes and weeks in all three ERG metrics (a-wave: p = 0.10; b-wave: p = 0.08; OP: p = 0.55), indicating the intermittent IOP elevation over a period of 2 months did not cause significant pre-loop retinal function alteration.

**Figure 6:**
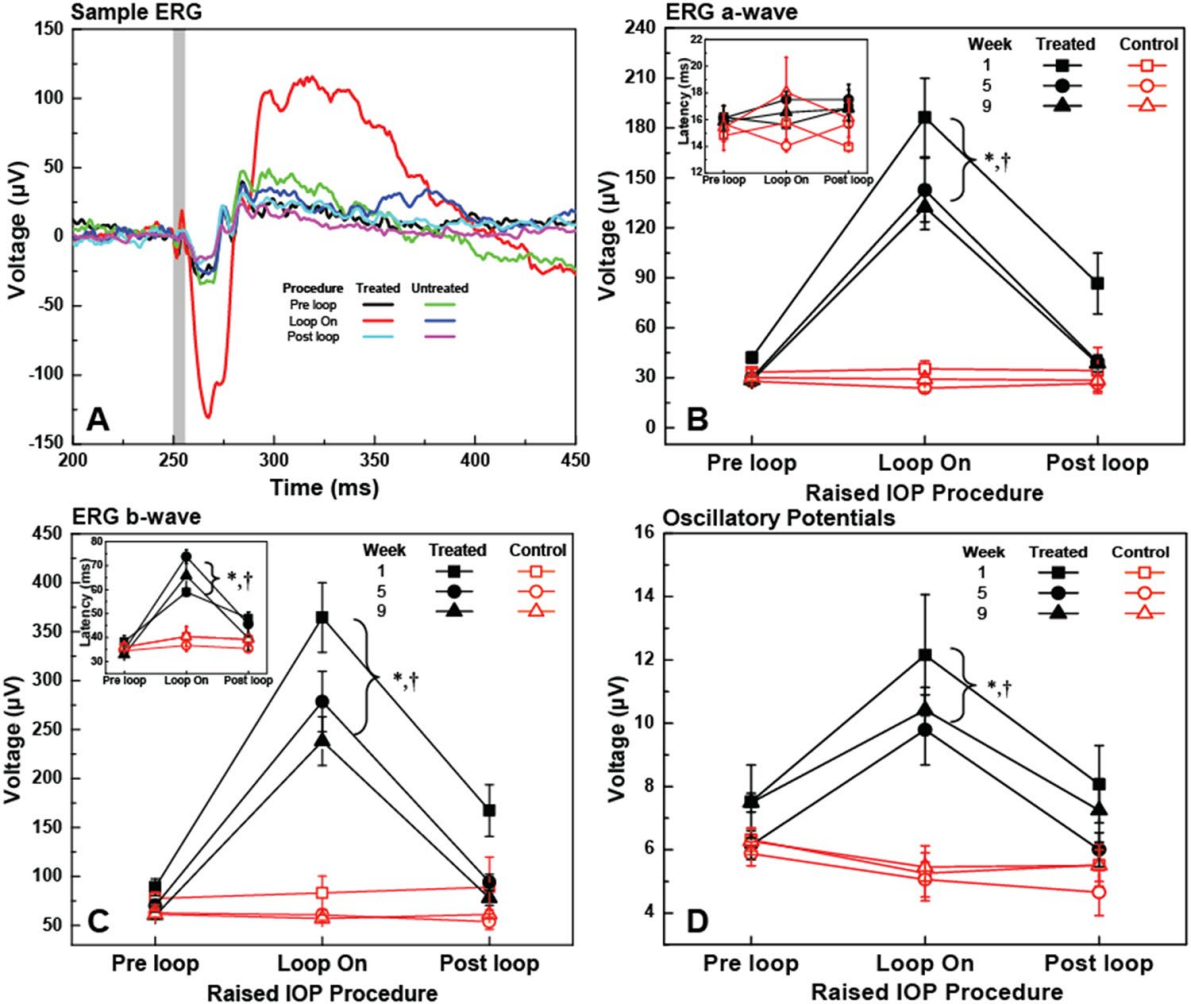
Representative ERG traces (1.14 log scotopic cd·s/m^2^) acquired from the treated and untreated eyes of a rat pre-, during, and post-loop wear at baseline (**A**). The vertical grey line marks the timing and duration of the visual stimulus. ERG a-wave amplitude (mean ± SE) (**B**) and latency (Inset in B) and ERG b-wave amplitude (**C**) and latency (Inset in C), and OP RMS (**D**) measured pre-, during, and post-loop wear at weeks 1, 5 and 9 of the study. *Significant difference relative to pre loop measurements in the treated eye averaged over weeks. †Significant difference relative to the loop wear measurements in the untreated eye averaged over weeks.

Figure 6A shows typical ERG recordings acquired pre-, during and post loop-wear in week 1 with stimulus intensity of 1.14 log scotopic cd·s/m^2^. Temporal increases in the ERG a-wave, b-wave and OP amplitudes were observed during acute elevated IOP in the treated eyes relative to the pre- and post-loop conditions. Specifically, averaged over all the weeks, the ERG a-wave, b-wave and OP amplitudes during loop wear in the treated eye were significantly larger than those measured pre-loop (all p < 0.01) or post-loop (all p < 0.01), while pre- and post-loop values were similar (all p > 0.05). The ERG metrics evaluated for both eyes were not significantly different for the pre- and post-loop procedures (all p > 0.05), while the interocular differences were significant during loop wear on the treated eye (all p < 0.01). The loop-associated enhancements in the treated eye decreased progressively from week 1 to week 9 of the study although not significantly (interaction between eyes, weeks and loop procedures; a-wave: p = 0.25, b-wave: p = 0.26, OP: p = 0.89; shown in Fig. 6B). The a-wave latencies did not change significantly as a function of the loop wear (p = 0.60) or across weeks (p = 0.33) or between eyes (p = 0.67; inset of Fig. 6B), and there is no interaction between eyes, weeks and loop procedures (p = 0.07; inset of Fig. 6C) in b-wave latency measurements. However, averaged over all weeks, the b-wave latencies increased significantly during loop wear in the treated eyes compared to the pre- and post-loop values in the same eye (loop vs pre, p < 0.01, loop vs post, p < 0.01) or relative to the untreated fellow eye values at the same time points (p < 0.01).

### Optic Nerve Histology

**Figure 7:**
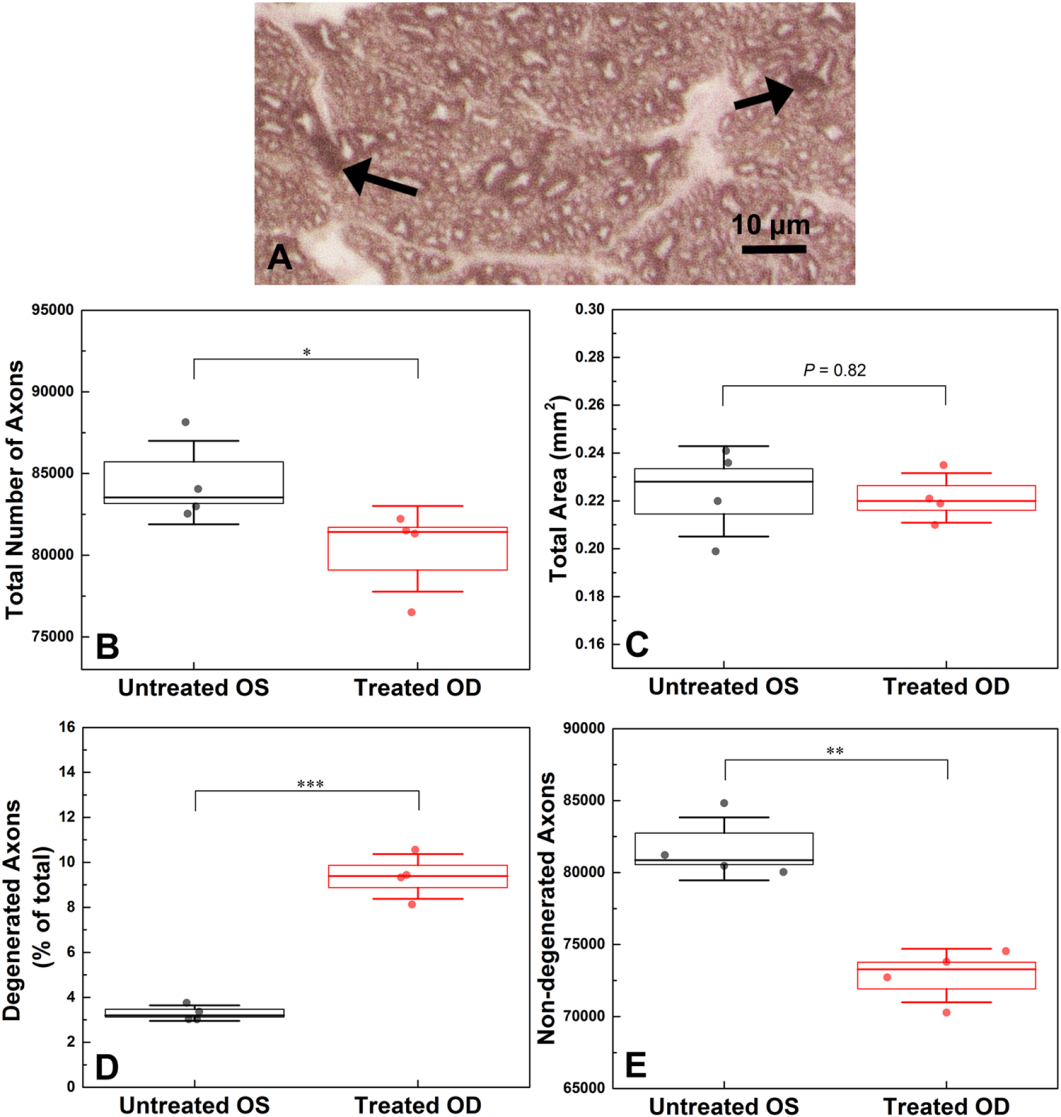
Histological image of an optic nerve axonal cross-sectional sample, obtained from a treated eye of one rat. Arrows: degenerative profile examples, scale bar = 10 µm (**A**). Boxplots for the total number of axons (**B**), the total axonal area (**C**), the percentage of degenerated axons (**D**) and the total number of non-degenerated axons (**E**). In each boxplot, line represents median, box represents SE and whiskers represent SD. *p < 0.05, **p < 0.05, ***p < 0.001.

Four animals from our study were adequately perfused postmortem in order to examine quantitatively the loss of axons in the optic nerve after 2 months of intermittent IOP elevation. The total number of axons (Fig. 7B) was significantly lower in the treated eyes (80,397 ± 2620) than the untreated eyes (84,440 ± 2553, p = 0.04), which constitutes ~5% loss of total number of axons. The cross-sectional area of the optic nerve was also assessed (Fig. 7C); however, no significant change was detected between the treated (0.22 ± 0.01 mm^2^) and untreated eyes (0.22 ± 0.02 mm^2^, p = 0.82).

The number of degenerating profiles in the treated optic nerves was 2.9 × higher than in untreated optic nerves (Fig. 7D) and the difference was significant (9.4 ± 1.0% vs 3.3 ± 0.4%, p < 0.01). Furthermore, in the cross-sectional area, the total number of non-degenerated axons (Fig. 7E) in the treated eyes (72,847 ± 1865) was significantly smaller than in the untreated eyes (81,648 ± 2179, p < 0.01).

## Discussion

For all measurement points of this chronic study, elevation of the IOP to a moderate level of ~35 mmHg for 1 h per day caused a temporary and reversible posterior displacement around the ONH, similar to what we observed in earlier acute IOP elevation studies with vascular loop^33,37^. The fact that the pre-loop and post-loop ONH depression measurements did not show any significant changes over the span of 8 weeks indicates that the mechanical deformation of the posterior segment structures caused by the chronic intermittent elevated IOP over a period of time of 8 weeks did not reach the ONH tissue elastic limit, and therefore did not cause any permanent morphological changes large enough to be detected in the unstressed state with the high resolution OCT system used in this study. However, we also observed that the ONH depression increased over time with chronic IOP elevation and was on average ~142% larger at week 9 compared to baseline (week 1). This increase in compliance during loop wear (increased mechanical load) suggests hypercompliance of the posterior segment tissue around the ONH. In a pathophysiological model discussing the relation between IOP-related stress/strain and ONH structure^38^, the ONH tissue was considered as a biomechanical component, and permanent deformation and surface hypercompliance of the ONH were suggested to be two separate outcomes of glaucomatous damage. The ONH hypercompliance was first reported by Burgoyne *et al*.^39^ in monkeys, and later confirmed by Ivers *et al*.^40^ and Yang *et al*.^41^. Although there is a morphological difference in the ONH between primates and rodents (mainly lack of laminar cribrosa in rodents), results from our chronic IOP study confirm the existence of peri-ONH and retinal tissue hypercompliance tissue in rats. Furthermore, we found that peri-ONH tissue hypercompliance increases over time without permanent unstressed deformation of the ONH after eyes were exposed to chronic, daily spikes of the IOP to moderate level of 35 mmHg and 1 h duration, indicating that the peri-ONH connective tissue elasticity perhaps may serve as an early biomarker for IOP-related glaucomatous risk prior to permanent structural change. This is supported by a recent study^42^ involving 104 human subjects that Sharma *et al*. reported glaucomatous eyes had significant reduction of neuroretinal rim width during acute IOP elevation, compared to the healthy and ocular hypertensive ones.

Recently, our group reported temporary, several-fold increase of the scotopic ERG amplitudes associated with acute, 1 h elevation of the IOP to 35 mmHg, that were observed consistently in different strains of rats using the vascular loop procedure^33,37^. Although in general, isoflurane anesthesia has been reported to suppress retinal function^43^ and therefore reduce the ERG amplitudes, a more recent study showed the persistence of increased ERG amplitudes during vascular loop wear using different types of anesthesia^44^. So far, the exact physiological origins of the several fold increase of the amplitudes of the ERG is not exactly known. A similar effect has been observed in other animal studies^45,46^, where ERG b-wave increased after acute IOP elevation with microbeads injection^45^, and after chronic IOP elevation with combined microbeads and sodium hyaluronate^46^. Results from our 2-month-long chronic study presented here, confirm that the amplitudes of both the ERG a-and b-waves are significantly higher during loop wear relative to the pre- and post-loop measurements.

While the mechanism(s) for the increase in the ERG amplitudes remains unknown, supranormal ERG amplitudes have been obtained in animals that have been exposed to nitric oxide donors^47^, adenosine^48^, dopamine blockers^49^ and perinatal lead^50^. In addition, loss of function of specific genes has been reported to result in elevated b-wave amplitudes; they include loss of mitochondrial ATP transporter in Ant1^−/−^ mice^51^, retinopathy, globe enlarged (*rge*) chicks younger than 6 weeks old^52^, and humans with genetic mutations in the *KCNV2* potassium channel genes^53^. Of the various conditions causing supranormal ERGs, nitric oxide enhancement of ERG amplitudes has been shown to be reversible, which suggests that it is a potential mechanism for our observations of the return of the ERG amplitudes to almost normal in the post-loop conditions.

To investigate the potential effect of the loop wear associated increase in the axial eye length on the ERG metrics, a simple mathematical model of the rat eye was created. As a first approximation, the rat eye has a slight elliptical shape at normal IOP^54^ with transverse length of 6.41 mm. From the SS-OCT data, we determined that for IOP of 35 mmHg, the lateral eye length decreased to 6.26 mm. The optical design of the custom visual stimulator integrated with the UHR-OCT imaging probe focused the stimulus light at the pupil plane with an angular spread of ±45°. Based on our simplified model of the rat eye and taking into account both the increase in the axial eye length and the change in the corneal curvature during loop wear, we determined that elevation of the IOP to 35 mmHg causes only ~11% increase in the illuminated area of the retina. However, since the energy of the visual stimulus beam was constant, the luminance level at the retina decreased with the increase of the illuminated area. The 11% increase in the visually stimulated area of the retina is insufficient to account for the approximately 4× increase in the amplitudes of the ERG metrics, indicating that the global eyeball shape change during loop wear is not the main cause for the observed ERG a- and b-wave amplitudes increase.

It is important to note that some research groups have observed decreases in the amplitudes of scotopic ERG a- and b-waves, and OPs with a constant IOP elevation in rodents, sustained for a prolonged period of time^10–12,14–17^. Specifically, Grozdanic, *et al*.^5^ reported ~25% and ~40% decrease in the scotopic ERG a-wave and b-wave amplitudes respectively in mouse retinas after 6 weeks of sustained IOP elevation using laser treatment of the trabecular meshwork and indocyanine green dye injection. Liu, *et al*.^9,10^ and Zhao, *et al*.^11^ used a circumlimbal suture to induce mild, constant IOP elevation. No significant changes in the ERG photoreceptor a-wave and bipolar b-wave amplitudes were present at two weeks, however, a significant reduction of the ERG photoreceptor a-wave and bipolar b-wave were observed as early as weeks 4^10^ and 8^9^ of the study, respectively. Moreover, OP amplitudes did not change significantly after 12 weeks of IOP elevation between 19–33 mmHg by circumlimbal suture while photoreceptor a-wave and bipolar b-wave reduced significantly at the same time point^11^. These studies showed that there are permanent structural and functional changes of the retina associated with constant moderately high IOP elevation over a prolonged period of time of at least 4 weeks. Based on these chronic studies and our own acute studies^33,37,44^, we expected that the amplitudes would decrease and/or decrease in their response to the elevated IOP over time. Although, the mean values for both weeks 5 and 9 loop-on data were lower, the data were not significantly different (Fig. 6B–D). One possible explanation for these results is that the integral exposure time of the retina to elevated IOP in our study was 48 h (8 weeks x 6 days/week × 1 h/day) which was significantly less than the total exposure time for other studies^10–12,14–17^ that demonstrated permanent structural and functional changes in the retina associated with elevated IOP, as cumulative IOP exposure time is closely related to the extent of retinal damage^55^. The fact that we observed a trend towards a decrease over time in the peak amplitudes of the ERG a-wave, b-wave and the OPs during the loop-on procedure (Fig. 6) suggests that there is a subtle change of the retinal function that is measurable when the retinal tissue is under IOP elevation. It is unknown whether significantly lower amplitudes would have been observed in animals that were exposed to intermittent IOP spikes for a duration longer than 8 weeks. However, the idea of a decrease in response to prolonged IOP elevation is supported by our observations that the optic nerve depression increases over time, in response to the IOP stress (Fig. 5).

While the exact mechanism of attenuated retinal function in response to IOP elevation is not fully understood, He *et al*.^56,57^ suggested that changes in the retinal function susceptibility to acute IOP elevations maybe related to chronic hypertension and -perfusion pressure. Moreover, several studies have demonstrated that IOP elevation could modify the retinal perfusion pressure and indirectly affect retinal funciton^49–51^. In our study, daily IOP elevations were conducted; therefore, it is hypothesized that animals were under repeated perfusion pressure modifications, which may contribute to the ocular function sensitivity change to the selective acute IOP elevation measurements. Since perfusion pressure measurements in awake rats during the imaging procedures were not possible due to the excessive tail motion, further studies need to be conducted to verify this hypothesis.

One limitation of this study is the group size. As the daily intermittent IOP elevation is labor-intensive, only six rats were included in this study. Conducting similar studies in the future over larger number of animals may provide more insight to the effect of intermittent IOP elevation on the retinal structure and function. Another limitation of this study is the inaccessibility of retinal ganglion cell function with the current OCT + ERG system. RGC function is insensitive to scotopic ERG components (a-wave, b-wave and OPs), and can be evaluated by scotopic threshold response (STR) with low stimulating illuminance (−6.06 to −3.04 log scotopic cd·s/m^2^) or pattern ERG; however, the custom LED stimulator in our OCT + ERG system was not designed to generate such low levels of illumination, and pattern ERG requires more sophisticated setup and is not available in the lab.

In conclusion, acute IOP spiking caused temporary changes in the retinal structure and function such as depression of the ONH, a relatively small increase of axial eyeball length and several fold increase in the ERG a- and b-wave amplitudes. Chronic, intermittent IOP spiking to moderate levels over a period of time of 8 weeks caused peri-ONH tissue hypercompliance without evidence of permanent posterior deformation or thinning of the GCL, GCC and total retina, or any significant change in the retinal function evaluated at normal IOP. However, chronic intermittent IOP elevation over a period of 8 weeks caused non-significant decrease in the ERG a- and b-wave amplitudes when measured during IOP elevation.
